# Increased frequency of intermetatarsal and submetatarsal bursitis in early rheumatoid arthritis: a large case-controlled MRI study

**DOI:** 10.1186/s13075-020-02359-w

**Published:** 2020-11-23

**Authors:** Yousra J. Dakkak, Ellis Niemantsverdriet, Annette H. M. van der Helm-van Mil, Monique Reijnierse

**Affiliations:** 1 Department of Rheumatology, Leiden University Medical Center, P.O. Box 9600, 2300 RC Leiden, The Netherlands; 2 Department of Rheumatology, Erasmus Medical Center, Rotterdam, The Netherlands; 3 Department of Radiology, Leiden University Medical Center, Leiden, The Netherlands

**Keywords:** Rheumatoid arthritis, Early arthritis, Healthy controls, Magnetic resonance imaging, Foot, Bursitis

## Abstract

**Background:**

The forefoot is a preferential location for joint and tendon sheath inflammation in rheumatoid arthritis (RA). It also contains bursae, of which the intermetatarsal bursae have a synovial lining. Some small imaging studies suggested that intermetatarsal bursitis (IMB) and submetatarsal bursitis (SMB) are involved in RA, but their association has not been thoroughly explored. Healthy control studies suggested that lesion size might be relevant. We studied the relation between IMB and SMB in early RA, compared to other arthritides and healthy controls, and the relevance of lesion sizes.

**Methods:**

Six hundred and thirty-four participants were studied: 157 consecutive patients presenting with early RA, 284 other arthritides, and 193 healthy controls. All underwent unilateral contrast-enhanced MRI of the forefoot at presentation. Two readers independently scored IMB and SMB and measured transverse and dorsoplantar diameters, blinded to clinical data. Subsequently, consensus was reached. Intra-reader ICC was 0.89. Logistic regression models were used, and test characteristics were calculated.

**Results:**

IMB and SMB associated with RA independent of each other (*P* < 0.001) and independent of age, gender, BMI, RA-MRI inflammation, and anti-CCP-antibodies (*P* = 0.041). Sensitivity for RA of IMB was 69%, and for SMB 25%. Specificity for IMB was 70% compared to other arthritides, and 84% compared to healthy controls. For SMB, this was 94% and 97% respectively. Regarding lesion size, the groups had considerable overlap: no cut-off size for RA could be distinguished with high sensitivity and specificity.

**Conclusion:**

Intermetatarsal and submetatarsal bursitis associated with early rheumatoid arthritis, contributing to the emerging evidence that inflammation of juxta-articular soft tissues is an early feature of RA.

## Key messages

- Intermetatarsal bursitis and submetatarsal bursitis associated with RA, and intermetatarsal bursitis had the highest sensitivity (69%).
- This contributes to the emerging evidence that in early RA extra-articular synovial inflammation is common.
- Regarding lesion size no cut-off for disease with high sensitivity and specificity could be distinguished.

## Background

Rheumatoid arthritis (RA) is a systemic autoimmune disease characterized by chronic, persistent inflammation of the synovium-lined joints and tendon sheaths [1, 2]. Preferential locations are the small joints of hands and feet. It has become apparent that early treatment with disease modifying anti-rheumatic drugs (DMARDs) improves disease outcome of RA patients [3]. Since prompt treatment has limited radiographic detectable damage, the European League Against Rheumatism (EULAR) recommends MRI for the early detection and follow-up of RA as it sensitively measures inflammation. According to the RA-MRI score (RAMRIS), inflammation is defined as synovitis, tenosynovitis, and bone marrow edema/osteitis [4, 5]. In the forefoot, however, imaging also reveals the presence and/or inflammation of bursae. So far, bursitis in the forefeet has gained little attention in RA literature.

Interestingly, naturally present bursae possess a synovial lining similar to synovial joints and tendon sheaths [6]. In the forefoot, intermetatarsal bursae are present, anatomically without a connection to the metatarsophalangeal (MTP) joints [7]. Two ultrasound studies have reported intermetatarsal bursitis (IMB) in patients with established RA [8, 9]. Additionally, one MRI study in 70 asymptomatic volunteers reported on fluid in the intermetatarsal spaces as a sign of IMB [10]. Gadolinium contrast enhancement was not used as a measure of inflammation. The authors suggested that a transverse diameter of ≤ 3 mm could be considered physiologic; however, these results have not been validated [10]. Thus, while data suggest that IMB may be associated with RA, its involvement in early disease has not been studied systematically.

In the subcutaneous fat, submetatarsal bursae that lack a synovial lining may develop due to high pressure and friction, leading to collagen degradation and localized fluid-collection that is visible on imaging as a sharply demarcated area in the submetatarsal space [11]. An ultrasound study reported submetatarsal bursitis (SMB) in patients with established RA [12]. However, this has also been observed in healthy controls. To the best of our knowledge, regarding IMB and SMB, no direct comparison has been made between RA and other arthritides, which is a comparison that is clinically relevant [13]. Also, no comparison has been made between RA and healthy controls, to analyze whether these findings could be normal.

Additionally, in the forefoot, Morton’s neuroma (MN) and diffuse submetatarsal alterations (DSMA) in the subcutis have been described. MN may emerge due to mechanical irritation, or secondary to IMB, and has been described in RA as well as asymptomatic volunteers [14–16]. DSMA consist of fibrosis or inflammatory tissue that may be induced by mechanical stress and may represent early stages in the development of SMB, particularly under the first and fifth metatarsal heads [17].

Overall, the current available literature does not give a fulfilling and thorough picture of the prevalence of IMB and SMB in RA patients. Therefore, this large cross-sectional MRI study was set up to study (1) the association of these lesions in RA, compared to other arthritides and to healthy controls, and (2) whether the size of these lesions can differentiate between diseases and healthy controls. Although not the main focus of this study, MN and DSMA were studied in subanalyses.

## Materials and methods

### Participants

Between June 2013 and March 2016, 447 consecutive patients newly presenting with clinical arthritis of a symptom duration < 2 years who were naïve to DMARDs were included in the Leiden Early Arthritis Cohort (EAC) [18]. The Leiden University Medical Center is the only rheumatology referral center within the Leiden area. Inclusion in the EAC of consecutive early arthritis patients has been part of regular care since 1993 [19]. Contrast-enhanced MRI of the forefoot was added to the protocol in June 2013. At baseline, swollen joint counts were performed, serum samples were obtained, and patients underwent MRI. Six MRI-examinations were excluded because of inhomogeneous fat suppression, 441 examinations remained (flowchart in Supplementary Figure S1). RA was defined as a clinical diagnosis of RA plus fulfillment of the 2010 RA criteria during the first year of follow-up [1]. The remaining patients received alternative diagnoses and were grouped together as ‘other arthritides’ (Table 1).

**Table 1:** Baseline characteristics of all participants

	RA	Other arthritides^1^	*P* value, RA vs other arthritides	Healthy controls	*P* value, RA vs healthy controls
	*n* = 157	*n* = 284		*n* = 193	
Clinical features					
Age, mean (SD)	59 (14)	56 (17)	0.07	50 (16)	< 0.001
Female, *n* (%)	109 (69)	158 (56)	0.005	136 (71)	0.83
BMI, mean (SD)	26 (5)	27 (4)	0.52	25 (4)	0.003
Symptom duration, in weeks, median (IQR)	10 (5–28)	8 (4–26)	0.13	NA	NA
Swollen joint count, median (IQR)	7 (2–11)	2 (1–4)	< 0.001	NA	NA
CRP, mg/L, median (IQR)	9 (4–26)	6 (3–16)	< 0.001	NA	NA
RF positive, *n* (%)	106 (68)	51 (18)	< 0.001	NA	NA
ACPA positive, *n* (%)	87 (59)	69 (25)	< 0.001	NA	NA
MRI features					
Mean number of lesions per patient, n (SD)					
Intermetatarsal bursitis	1.6 (1.4)	0.6 (1.0)	< 0.001	0.2 (0.7)	< 0.001
Submetatarsal bursitis	0.4 (0.8)	0.07 (0.3)	< 0.001	0.04 (0.2)	< 0.001
Morton’s neuroma	0.4 (0.6)	0.06 (0.3)	< 0.001	0.03 (0.2)	< 0.001
Diffuse submetatarsal alterations	0.5 (1.1)	0.3 (0.9)	0.068	0.3 (0.9)	0.092

*RA* rheumatoid arthritis, *SD* standard deviation, *BMI* body mass index, IQR interquartile range, *CRP* C-reactive protein, *RF* rheumatoid factor, *ACPA* anti-citrullinated peptide antigen (anti-CCP), *MRI* magnetic resonance imaging, *NA* not applicable

^1^This group included the following diagnoses: unclassified arthritis (*n* = 148), psoriatic arthritis or spondyloarthritis (*n* = 45), inflammatory osteoarthritis (*n* = 23), reactive arthritis (*n* = 7), crystal arthropathy (*n* = 21), remitting seronegative symmetrical synovitis with pitting edema (*n* = 12), and other diagnoses (*n* = 28)

Healthy controls were recruited by advertisements in local newspapers and websites, as reported previously [20]. Participants had no history of inflammatory rheumatic disease, no joint symptoms during the last month, and no arthritis at physical examination.

The EAC and healthy control study were approved by the local medical ethics committee (approval numbers P10.108 and P17.261). Informed consent was obtained.

### MRI scanning

Patients and symptom-free persons were scanned according to our routine MRI protocol as described in Supplementary Data S1. They underwent unilateral MRI of the hand and forefoot of the more painful side, or the dominant side in case of symmetrical symptoms, ≤ 2 weeks after the first presentation and before start of DMARDs. In symptom-free persons, the dominant side was scanned. A musculoskeletal extremity 1.5-T MRI unit (Oni; GE Healthcare, Madison, WI, USA) was used with a 145-mm coil. Acquired sequences for the forefoot after intravenous injection of gadolinium contrast included: axial T1-weighted fast spin-echo with fat suppression (repetition time ms/echo time ms 700/9.5; acquisition matrix 364 × 224, echo train length 2) and coronal T1-weighted fast spin-echo with fat suppression (540/7.5; acquisition matrix 320 × 192, echo train length 2). Field-of-view was 140 mm, slice thickness 3 mm, and slice gap 0.3 mm for both planes. Axial sequences had 14 slices, and coronal sequences 20 slices.

### MRI evaluation

#### Anatomy and scoring system

**Fig. 1:**
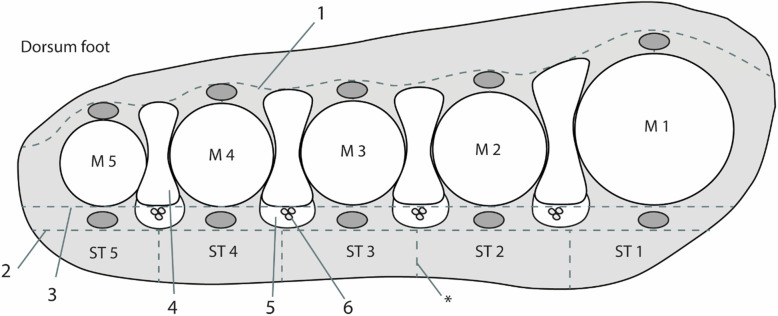
Schematic illustration of the forefoot at the metatarsal heads with intermetatarsal and submetatarsal spaces. The intermetatarsal space is demarcated at the dorsal side by the deep dorsal aponeurosis (1) and at the plantar side by the superficial transverse metatarsal ligament (2). The deep transverse metatarsal ligament (3) divides the intermetatarsal space into a superior (4) and inferior part (5), respectively, containing the bursa and neurovascular bundle (6). The submetatarsal spaces (ST) are located in the subcutis and are artificially bordered by the midline of the intermetatarsal space (*). M, metatarsal heads. Gray ovals represent extensor and flexor tendons of the forefoot

The forefoot was divided into four intermetatarsal and five submetatarsal spaces (Fig. 1) [21]. The intermetatarsal space is dorsally bound by the deep dorsal aponeurosis and plantar by the superficial transverse metatarsal ligament [6, 7]. It is divided into a superior and inferior level by the deep transverse metatarsal ligament. The intermetatarsal bursae lie in the superior intermetatarsal spaces [6], the neurovascular bundle, from which MN originates, lies in the inferior intermetatarsal space and has a close cohesion with the synovial lining of the intermetatarsal bursae [6, 7]. The submetatarsal spaces lie in the subcutis, plantar to the superficial transverse metatarsal ligament, and extend until the epidermis. We used the following definitions:

- Intermetatarsal bursitis (IMB). Contrast enhancement in the superior intermetatarsal space with or without rim enhancement
- Submetatarsal bursitis (SMB). A sharply demarcated area with contrast enhancement in the submetatarsal space with or without rim enhancement
- Morton’s neuroma (MN). An isolated spindle-shaped lesion in the inferior intermetatarsal space with or without contrast enhancement, without rim enhancement continuous with IMB [10]
- Diffuse submetatarsal alterations (DSMA). An unsharply defined area in the submetatarsal space with diffuse contrast enhancement

Lesions had to be visible on two consecutive slices in both axial and coronal planes. Besides scoring for presence, the transverse and dorsoplantar diameter of IMB, SMB, and MN were measured in millimeters on coronal images. DSMA was not measured as, per definition, it has no sharply demarcated borders.

#### Scoring

Two raters (a musculoskeletal radiologist with 23 years of experience, and a trained MD with 2 years of experience in RAMRIS scoring and has scored > 400 MRIs according to this system during a training period of several months prior to evaluating the MRIs that are part of this study) [22, 23], who were blinded to all clinical data, independently scored the MRIs. The two raters determined the final scores by consensus: a lesion was only regarded to be present if both readers agreed on this. First, all early arthritis patients were scored, followed by healthy controls. To exclude observer bias introduced by knowing that images belonged to healthy controls, MRIs of healthy controls were randomly mixed with 20 MRIs from EAC patients. Based upon these 20 patients that were scored twice an intra-reader intraclass correlation coefficients (ICC) was determined that was 0.89.

In addition, to explore whether intermetatarsal and submetatarsal lesions were associated with RA independent of RAMRIS inflammation, the MRIs were also scored for synovitis, tenosynovitis, and osteitis at MTP joints in line with the RAMRIS by two independent readers, also blinded to clinical data, as is described in Supplementary Data S1 [22–25]. RAMRIS inflammation was considered present when synovitis, tenosynovitis, and/or osteitis were scored as ≥ 1 by both readers.

### Analyses

IMB and SMB were studied in RA patients, other arthritides, and healthy controls. First, as patients could have more than one lesion, e.g., one patient could have IMB at more than one location, the mean number of lesions in RA patients was compared to patients with other arthritides, and to healthy controls using Mann-Whitney *U* tests.

Next, the data was dichotomized for presence per lesion: e.g., whether a patient had IMB at any location. Logistic regression analysis was used to compare RA to other arthritides and to healthy controls. Multivariable models corrected for the simultaneous occurrence of the different lesions and for age, gender, BMI, presence of RAMRIS inflammation, and anti-citrullinated peptide antibodies (anti-CCP), as these may be important in the relation between these lesions and RA [26, 27]. Test characteristics for RA were determined. Heatmaps were plotted with the percentage of participants with a lesion per location. Finally, measured transverse and dorsoplantar diameters were plotted to assess whether a cut-off for disease could be observed.

As subanalyses the analyses were repeated for MN and DSMA.

Calculations were performed with SPSS Statistics, version 23.0; IBM, Armonk, NY. *P* < 0.05 was considered statistically significant.

## Results

### Patient characteristics

Of the 441 consecutively included EAC patients, 157 were classified as early RA, 284 patients had other arthritides (Table 1). One hundred and ninety-three healthy controls were recruited. RA patients were predominantly female (69%) and had a median symptom duration of 10 weeks (interquartile range: 5–28).

RA patients had a higher number of IMB and SMB lesions per patient compared to other arthritides and to healthy controls (all *P* < 0.001) (Table 1). Next, the presence of a lesion was dichotomized as described in the Methods.

### Association of inter- and submetatarsal bursitis with RA

The number of participants with lesions is given in Table 2. RA patients more often had IMB and SMB than other arthritides (all *P* < 0.001).

**Table 2:** The association of intermetatarsal and submetatarsal lesions with early RA compared to other early arthritides

	Participants with MRI features, *n* (%)		Univariable analyses		Multivariable analysis^1^		Multivariable analyses^2^	
	RA	Other arthritides	*OR* (95% CI)	*P* value	*OR* (95% CI)	*P* value	*OR* (95% CI)	*P* value
Intermetatarsal bursitis	109 (69)	84 (30)	5.4 (3.5–8.3)	< 0.001	4.5 (2.7–7.8)	< 0.001	3.7 (2.1–6.6)	< 0.001
Submetatarsal bursitis	39 (25)	17 (6)	5.2 (2.8–9.5)	< 0.001	2.2 (1.03–4.5)	0.041	2.3 (1.1–4.8)	0.031
Morton’s neuroma	30 (19)	10 (4)	6.7 (3.2–14.2)	< 0.001	–	–	3.1 (1.3–7.7)	0.012
Diffuse submetatarsal alterations	36 (23)	45 (16)	1.6 (0.9–2.6)	0.067	–	–	0.9 (0.5–1.8)	0.86

The results of logistic regression analyses are presented. *RA* rheumatoid arthritis, *OR* odds ratio, *CI* confidence interval

^1^Multivariable model including intermetatarsal bursitis, submetatarsal bursitis, age, gender, anti-CCP, and RAMRIS inflammation (defined as the presence of synovitis, tenosynovitis, and/or osteitis)

^2^Multivariable model including intermetatarsal bursitis, submetatarsal bursitis, Morton’s neuroma, diffuse submetatarsal alterations, age, gender, BMI, anti-CCP antibodies, and RAMRIS inflammation

Since IMB and SMB were both associated with RA, and in addition age, gender, BMI, RAMRIS inflammation, and anti-CCP-antibodies may be important in the relation between the lesions and RA, a multivariable model was performed that included both lesions and these clinical parameters [26, 27]. IMB and SMB remained associated with RA independent of these factors (Table 2); the effect size was largest for IMB (OR 4.5, 95%CI 2.7–7.8).

The analyses were repeated comparing RA patients to healthy controls, revealing similar results (Supplementary Table S1).

### Test characteristics of intermetatarsal and submetatarsal bursitis for RA

Next, test characteristics were determined for IMB and SMB (Supplementary Table S2). Sensitivity for IMB was 69%, and for SMB 25%. Specificity of IMB compared to other arthritides was 70%, and compared to healthy controls 84%. For SMB, this was 94% and 97%, respectively.

### Heatmap of lesions for RA, other arthritides, and healthy controls

**Fig. 2:**
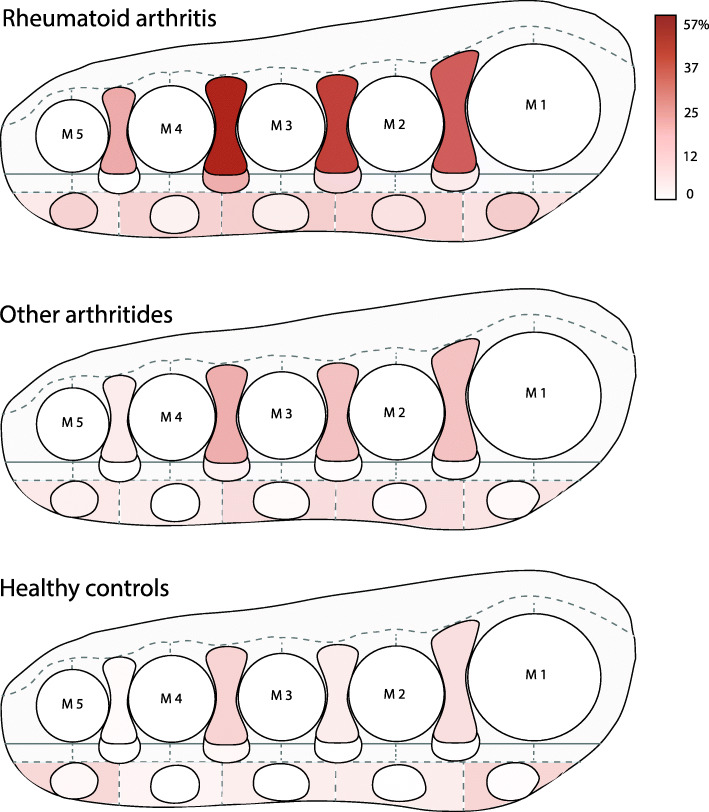
Heatmap of intermetatarsal bursitis, submetatarsal bursitis, Morton’s neuroma, and diffuse submetatarsal alterations for every population. Schematic illustration in coronal view of the frequency of lesions in each compartment of the forefoot at the level of the metatarsal heads (see also supplementary Table S3). The frequency of the lesions (% of participants in the respective group) is represented by an increase in color intensity. The compartments are defined in Fig. 1. Mortons neuroma (MN) is demarcated plantar to intermetatarsal bursitis (IMB). In the subcutis, submetatarsal bursitis (SMB) is illustrated as a demarcated oval. The remainder of the subcutis represents diffuse submetatarsal alterations (DSMA). IMB, SMB, and MN are most frequently seen in RA. The second and third IMB are preferred locations whereas the fourth is the least involved. In the subcutis of RA patients, DSMA is seen under MTP 2, 3, and 4, whereas in healthy controls, this is seen under MTP 1 and 5. SMB dominates under MTP 1 and 5 in RA patients. M, metatarsal heads

The distribution of the lesions was plotted in heatmaps (Fig. 2, Supplementary Table S3). In RA, IMB affected the 3rd intermetatarsal space most (57% of patients). Regarding SMB, the first and fifth submetatarsal space were most affected: in 13% and 12% of patients, respectively.

**Fig. 3:**
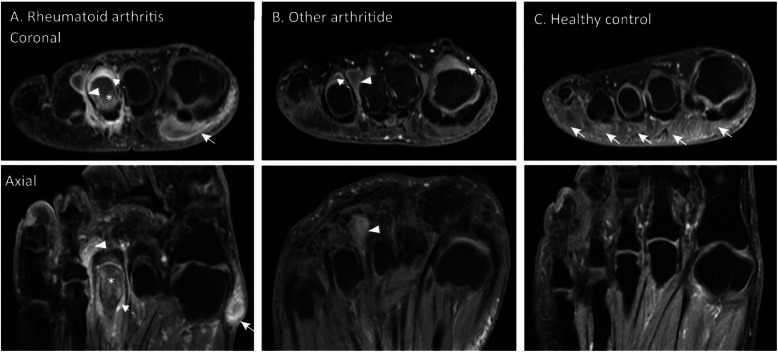
MR examples of intermetatarsal and submetatarsal bursae. Coronal and axial fat suppressed T1-weighted FSE gadolinium-enhanced images of the forefoot at the level of the metatarsal heads. **a** Female participant with RA (age 61 years) with intermetatarsal (IMB) and submetatarsal bursitis (SMB). IMB in the 3rd intermetatarsal space (arrowhead) with peripheral enhancement protruding dorsal (dumbbell shape) and plantar (teardrop shape) of the metatarsal heads. Peripheral enhancement of a mass in the first submetatarsal space, consistent with SMB (white arrow). Synovitis of MTP 3 (dotted arrow), as well as osteitis in the head of the third metacarpal bone and proximal phalanx (*). **b** Female participant with another arthritide (diagnosis of viral reactive arthritis, age 34 years) and IMB at the 3rd intermetatarsal space (arrowhead) with dorsal protrusion. Additional synovitis of MTP 1 and 4 (dotted arrows). **c** Female healthy control (age 50 years) with diffuse submetatarsal alterations (DSMA) in all submetatarsal spaces, predominantly visible at the 1st, 2nd, and 4th submetatarsal spaces (arrows). Intense linear contrast enhancement at the 2nd intermetatarsal space is consistent with a small vessel on the consecutive slices (not shown), there is no IMB visible on the axial images

Similarly, in other arthritides and healthy participants, the 3rd intermetatarsal space was affected by IMB most often, but with lower frequency (20% and 11% respectively). For SMB at the first and fifth submetatarsal space this was 2% and 4%, respectively, in other arthritides and 1% and 2% in healthy controls. MRI-examples are given in Fig. 3.

### Transverse and dorsoplantar diameter of lesions for patients with rheumatoid arthritis, other arthritides, and healthy controls

**Fig. 4:**
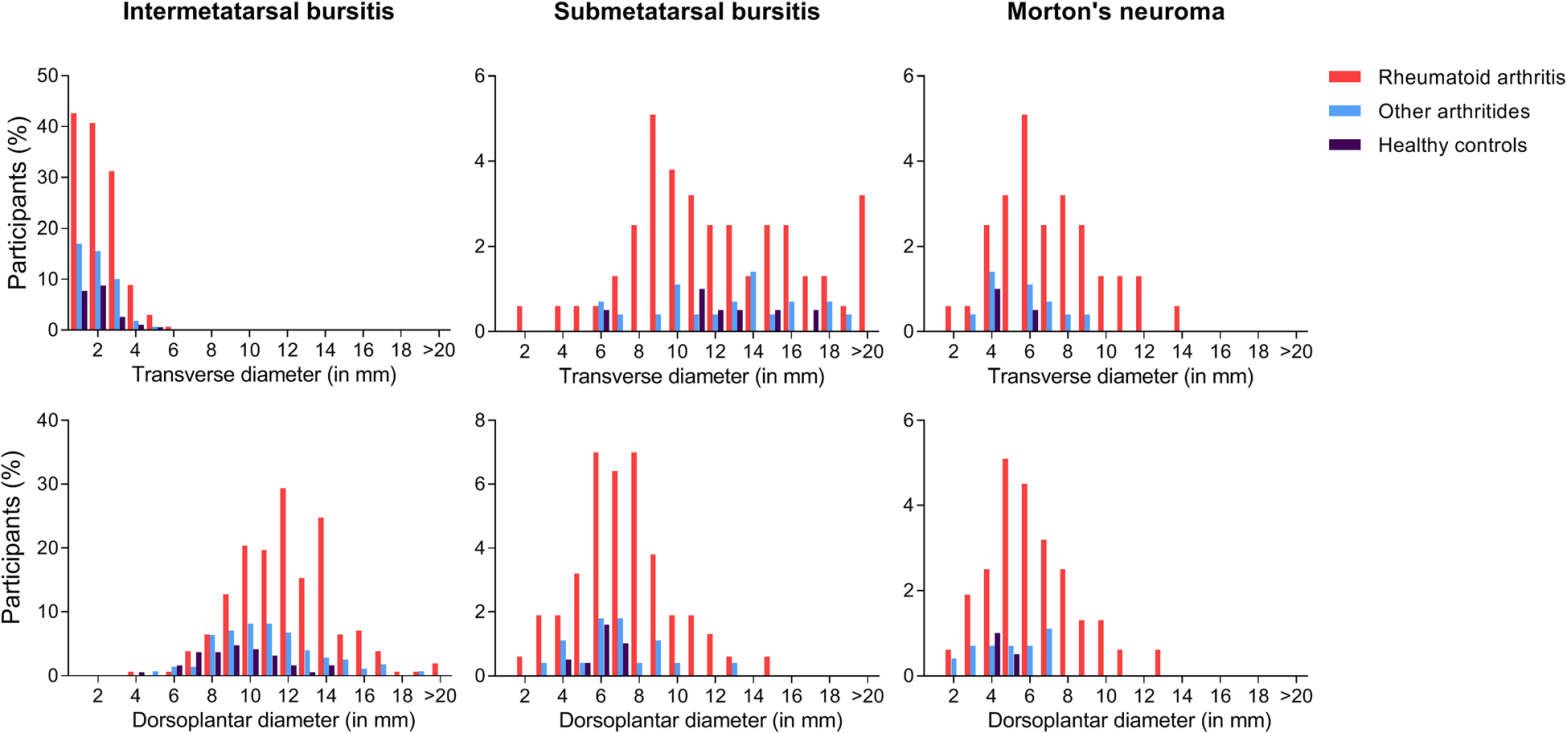
Distribution of transverse and dorsoplantar diameters (mm) of intermetatarsal bursitis, submetatarsal bursitis, and Morton’s neuroma. The *x*-axis displays the diameter (in mm), the *y*-axis the percentage of participants with the corresponding diameter. Participants could have more than one lesion; therefore, the percentage of participants with a lesion does not add up to the total amount of participants. The metatarsal bones limit the IMB in the transverse plane, squeezing the bursa outwards in the dorsoplantar plane. The SMB expands more in the transverse than dorsoplantar plane in the subcutis. The detected MN are predominantly round and larger than 5 mm

Next, the transverse and dorsoplantar diameters of IMB and SMB were measured (in mm) and plotted in histograms (Fig. 4). We evaluated cut-offs based upon the histograms (Supplementary Table S4).

First the transverse diameter of IMB was studied. From the histograms it was apparent that lesions ≥ 4 mm were infrequent in other arthritides (3% of patients) and healthy controls (2%). We studied whether this could be used as a cut-off for RA: however lesions ≥ 4 mm were also less frequent in RA (13%). In all groups the majority of lesions were ≤ 3 mm: 68% of RA patients had a lesion ≤ 3 mm, compared to 29% other arthritides and 15% of healthy controls. Hence, a cut-off of 3 mm increased specificity to 97% compared to other arthritides and 98% to healthy controls, at the cost of a decrease in sensitivity to 13%.

Theoretically, IMB may be limited by the metatarsal heads in its ability to distend transversely and may, therefore, upon inflammation, distend dorsoplantar. A dorsoplantar diameter of IMB ≥ 15 mm was infrequent in the control groups (Fig. 4). Taking ≥ 15 mm as a cut-off increased specificity for RA compared to other arthritides (95%) and healthy controls (100%). Sensitivity, however, decreased to 16%.

Similarly, regarding SMB no cut-off could be determined that did not lead to a substantial decrease in sensitivity for RA (Supplementary Table S4).

### Subanalyses for MN and DSMA

MN was associated with RA compared to other arthritides in univariable and multivariable analyses that included IMB, SMB, MN, DSMA and clinical parameters (*P* < 0.001 and *P* = 0.012 respectively). DSMA was not associated (*P* = 0.069 and *P* = 0.86, respectively) (Table 2). Similar results were obtained when compared to healthy controls (Supplementary Table S1).

The test characteristics for MN were determined and were as follows: sensitivity 19%, and specificity 96% compared to other arthritides and 98% compared to healthy controls. For DSMA this was not determined as it was not associated with RA.

MN and DSMA were incorporated in the heatmaps in Fig. 2 (Supplementary Table S3 presents the exact frequency per location). MN occurred most frequently in the third intermetatarsal space (20% of RA patients). DSMA in the first and fifth submetatarsal space were relatively more common in healthy controls (both locations 10%).

Regarding lesion size for MN, a transverse diameter of ≥ 7 mm was infrequent in control groups (Fig. 4): using this cut-off increased specificity for RA, however sensitivity decreased to 12%. Additionally, literature suggests a cut-off of ≥ 5 mm, this decreased sensitivity to 16% [10, 28]. Similarly, for the dorsoplantar diameter, using a ≥ 4 mm cut-off based on Fig. 4 decreased sensitivity to 13% (Supplementary Table S4). DSMA was not measured as, per definition, it has no sharply demarcated borders.

## Discussion

This cross-sectional study aimed to explore MRI-detected IMB and SMB in consecutive patients presenting with early RA, compared to other arthritides and healthy controls. We observed that both IMB and SMB were associated with and specific for RA (specificity ranging from 70 to 97%). In addition, we studied whether lesion size might be relevant and found considerable overlap in size between the groups; therefore, no cut-off for RA could be distinguished with high sensitivity and specificity. To our knowledge, our study is the largest to date to systematically study involvement of bursae in the forefoot in RA.

IMB results from inflammation of a naturally present, synovium-lined, structure [6]. Since RA is a disease of the synovium, the bursae might be a primary focus of disease, previously unnoticed. It can be hypothesized that IMB occurs secondary to concomitant arthritis; however, there is no anatomical connection between intermetatarsal bursae and MTP joints and in our study IMB associated with RA independent of the presence of RAMRIS inflammation, defined as synovitis, tenosynovitis, and osteitis [7].

In the plantar subcutaneous fat of the forefoot SMB was scored as sharply demarcated submetatarsal areas of contrast enhancement with or without rim enhancement that may develop secondary to mechanical loading [17]. These non-native (adventitious) bursae form at sites of friction but have imaging features similar to native bursae [29].

In addition MN and DSMA were scored, of which MN was also associated with RA. This association remained present after correcting for age, gender, and BMI; features that may relate to other pathologies predisposing to MN. Thus, MN seems to be increased in RA. Within RA, IMB has previously been suggested as a cause of MN [14, 30]. Inflamed bursae may irritate the common plantar digital nerve [6, 30], with secondary MN formation [7, 30]. This may explain why MN mostly occurred at locations where IMB was most frequent (second and third intermetatarsal spaces). For the definition of MN, besides the shape and location, it was important in our study that the lesion did not have any rim enhancement that was continuous with the IMB. This might be especially challenging at the second and third intermetatarsal spaces, where enlarged intermetatarsal bursae can extend below the deep transverse metatarsal ligament towards the neurovascular bundle [10]. In the literature the prevalence for MN varies between studies, from 15% in symptomatic volunteers to 54% in asymptomatic volunteers [10, 16, 31]. An explanation for the low frequency of 20% in our study could be the challenging differentiation between MN and IMB and potential co-existence of the two, especially for smaller MN. The limited MRI protocol, without T1 and fluid-sensitive sequences, may underscore fibrous MN [32]. Therefore, no definitive conclusion regarding the frequency of MN can be drawn from our study. We do not believe this undermines the association that was found of MN with RA, as the protocol restriction applies for all study groups.

DSMA, interestingly, occurred in healthy controls specifically under the first and fifth metatarsal heads. These locations are consistent with a previous study and are considered a normal finding based on mechanical loading [17]. In RA, DSMA occurred predominantly under MTP 2, 3, and 4, whereas under MTP 1 and 5 SMB were seen (Fig. 2).

It is difficult to compare our findings to previous studies in RA, as they utilized ultrasound and included patients with established RA receiving treatment rather than early disease [8, 33].

The intermetatarsal space is limited in the transverse plane by the metatarsal bones. Thus, bursitis is squeezed either dorsal and or plantar, especially at the second and third intermetatarsal spaces, respectively called a dumbbell or a teardrop phenomenon (Fig. 1), explaining the larger dorsoplantar diameter that is found (Fig. 4) [7, 21].

Our study has some limitations. First, the scoring method is not validated; therefore, reading was done by consensus rather than by independent readers, as this may be necessary in the setting of preliminary findings [34]. However, for determining the validity of an outcome measure, it is crucial to demonstrate the reliability of scoring between independent readers [34, 35]. The Intra-reader ICC, however, was determined and was reassuring. Second, our MRI protocol contained no fluid-sensitive sequences, as the protocol was originally intended to score RAMRIS inflammation. Therefore, small amounts of fluid in the bursae may have remained undetected, leading to an underestimation of observed lesions. Nevertheless, the use of contrast enhancement might be a strength, which previous studies in early arthritis found essential for optimal assessment of MRI-detected synovitis and tenosynovitis [25, 36]. Because intermetatarsal bursae have a synovial lining, we assumed that contrast enhancement would increase sensitivity of synovial hypertrophy in (active) bursitis. Thus, although theoretically small amounts of fluid in a bursa may be missed without a fluid-sensitive sequence, this could be normal in a bursa and rarely occurs at this location without enhancing synovial hypertrophy [10, 37]. The greater prevalence of contrast-enhancing (teno-)synovitis might be expected to occur in RA. To account for this we included RAMRIS inflammation in our multivariable model. Third, although the lesions were associated with RA independent of the factors that were adjusted for, additional factors might be of influence to the occurrence of these lesions, such as physical activity and type of shoes that were unaccounted for and may potentially be a source of bias. Also, we did not include a comparison with weight-bearing radiographs [38, 39]. Finally, our study was cross-sectional in nature. Thus, although our study suggests that these lesions may aid the clinician as a (differential) diagnostic tool, prospective studies are warranted to further establish the diagnostic relevance of these lesions.

## Conclusion

IMB and SMB are both associated with RA. As IMB has a synovial lining, these results contribute to the emerging evidence that in early RA, besides intra-articular synovitis, juxta-articular synovial inflammation is common. Previously, tenosynovitis was reported [18, 36], and now IMB as well. The current findings pose questions on whether the bursitis correlated to symptoms, responds to therapy, or has diagnostic value, e.g., in predicting RA development in the early phases of disease.

## Supplementary Information

**Additional file 1.**

## Data Availability

The datasets analyzed during the current study are available from the corresponding author on reasonable request.

## Abbreviations

RA: Rheumatoid arthritis
IMB: Intermetatarsal bursitis
SMB: Submetatarsal bursitis
MRI: Magnetic resonance imaging
DMARDs: Disease modifying anti-rheumatic drugs
EULAR: European League Against Rheumatism
RAMRIS: RA-MRI score
MTP joint: Metatarsophalangeal joint
MN: Morton’s neuroma
DSMA: Diffuse submetatarsal alterations
EAC: Early Arthritis Cohort
ICC: Intraclass correlation coefficients
anti-CCP: Anti-citrullinated peptide antibodies
