# Supplementary material for: Increased frequency of intermetatarsal and submetatarsal bursitis in early rheumatoid arthritis: a large case-controlled MRI study
Source: Arthritis Res Ther. 2020 Nov 23;22:277. doi: 10.1186/s13075-020-02359-w (PMC7684940; doi:10.1186/s13075-020-02359-w)
Supplement: Supplementary file 1 — Additional file 1. [file 13075_2020_2359_MOESM1_ESM.docx]

# SUPPLEMENTARY FILES

# Supplementary Figure S1. Flow chart of participants.


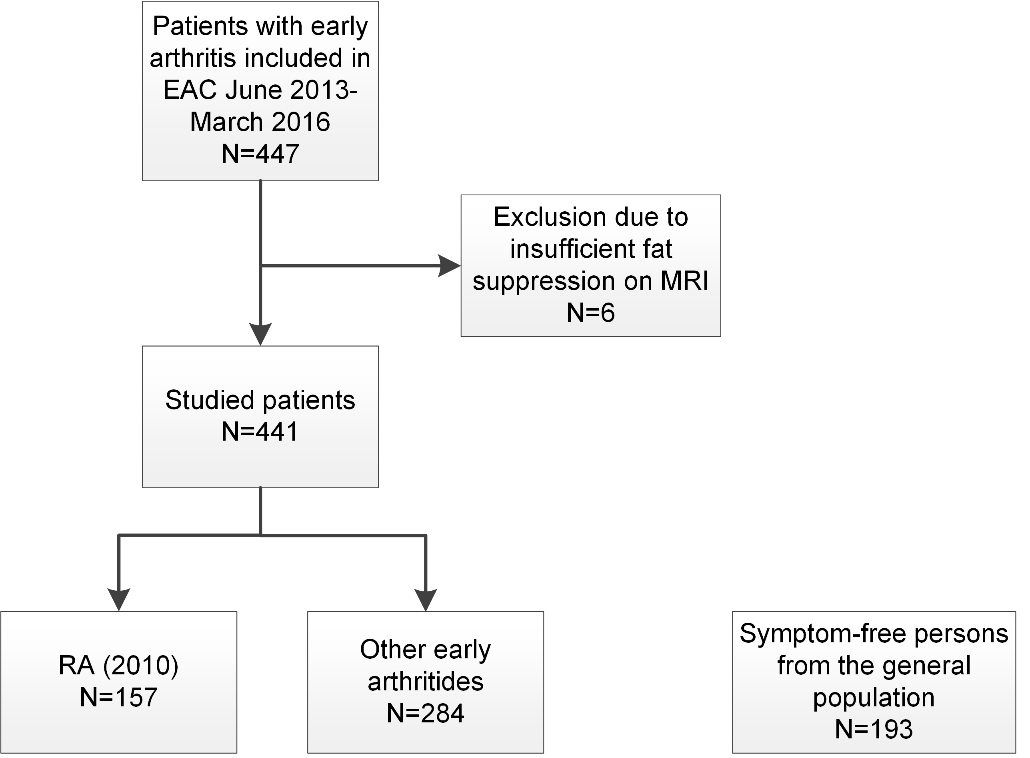


RA was defined according to clinical diagnosis of RA plus fulfilment of the 2010 classification criteria. The ‘other early arthritides’ included the following diagnoses: unclassified arthritis (n=148), psoriatic arthritis or spondyloarthritis (n=45), inflammatory osteoarthritis (n=23), reactive arthritis (n=7), crystal arthropathy (n=21), remitting seronegative symmetrical synovitis with pitting oedema (n=12) and other diagnoses (n=28).

This flowchart is similar as described in Dakkak, *et al* 2020.[8]

# Supplementary Data S1. Scoring of RA-MRI-inflammation: synovitis, tenosynovitis and osteitis

Synovitis and bone marrow edema at MTP-joints were scored in line with the validated Outcome Measures in Rheumatology Clinical Trials Rheumatoid Arthritis Magnetic Resonance Imaging Scoring-system (OMERACT RAMRIS).[1] For tenosynovitis, the score as described by Haavardsholm *et al* was applied to the extensor and flexor tendons of the MTP-joints.[2]

The synovitis score (range 0-3) was scored based on the volume of enhancing tissue in the synovial compartment (none, mild, moderate, severe). The tenosynovitis score (range 0-3) was based on the thickness of peritendinous effusion or synovial proliferation with contrast enhancement (normal, <2mm, 2-5mm, >5mm). Bone marrow edema was scored on a 0-3 scale based on the affected volume of the bone (no osteitis, >0-33%, >33-66%, >66%).

For this, each MRI was scored blinded to clinical data by two independent readers, both physicians and doctoral candidates in the field of rheumatology and radiology, with 1-2 years of experience with the scoring systems. Interreader and intrareader ICCs were generally ≥0.90.[3]

MRI-inflammation was considered present when synovitis, tenosynovitis and/or bone marrow edema were scored as ≥1 by both readers.

Bone marrow edema was assessed on a contrast-enhanced T1-weighted fat-suppressed sequence, as its use for depicting bone marrow edema is agreed on by the European Society of Musculoskeletal Radiology (ESSR) and previous studies have demonstrated that it has a strong correlation with the T2-weighted fat suppressed sequence that is advised by the RA-MRI-inflammation score.[4-7]

# Supplementary Table S1. Results of logistic regression analyses for the association of intermetatarsal and submetatarsal lesions with early RA compared to healthy controls

|  | Participants with MRI-features, n (%) | | Univariable analyses | |
| --- | --- | --- | --- | --- |
|  | RA | Healthy controls | *OR (95% CI)* | *P-value* |
| Intermetatarsal bursitis | 109 (69) | 31 (16) | 11.9 (7.1-19.8) | <0.001 |
| Submetatarsal bursitis | 39 (25) | 3 (2) | 15.6 (4.7-52) | <0.001 |
| Morton’s neuroma | 30 (19) | 6 (3) | 10.3 (4.2-25.1) | <0.001 |
| Diffuse submetatarsal alterations | 36 (23) | 31 (16) | 1.6 (0.9-2.7) | 0.11 |
|  | Multivariable analysis^1^ | | Multivariable analyses^2^ | |
|  | *OR (95% CI)* | *P-value* | *OR (95% CI)* | *P-value* |
| Intermetatarsal bursitis | 6.9 (3.9-12.5) | <0.001 | 5.5 (3.0-10.2) | <0.001 |
| Submetatarsal bursitis | 3.7 (1.2-11.3) | 0.023 | 3.7 (1.2-11.7) | 0.023 |
| Morton’s neuroma | - | - | 4.0 (1.0-15.5) | 0.047 |
| Diffuse submetatarsal alterations | - | - | 1.0 (0.5-2.3) | 0.93 |

RA: rheumatoid arthritis; OR: odds ratio; CI: confidence interval. ¹Multivariable model including intermetatarsal bursitis, submetatarsal bursitis, age, gender, anti-CCP and RAMRIS-inflammation (defined as the presence of synovitis, tenosynovitis and/or bone marrow edema). ²Multivariable model including intermetatarsal bursitis, submetatarsal bursitis, Morton’s neuroma, diffuse submetatarsal alterations, age, gender, BMI, anti-CCP antibodies and RAMRIS-inflammation

# Supplementary Table S2. Test characteristics for intermetatarsal and submetatarsal lesions for RA

|  | RA | RA vs. other arthritides | RA vs. healthy controls |
| --- | --- | --- | --- |
|  | Sensitivity | Specificity | Specificity |
| Intermetatarsal bursitis | 69 (62-72) | 70 (65-75) | 84 (78-88) |
| Submetatarsal bursitis | 25 (19-32) | 94 (91-96) | 97 (93-99) |
| Morton’s neuroma | 20 (14-26) | 96 (94-98) | 98 (96-100) |

RA: rheumatoid arthritis; Values are depicted in % with their corresponding 95% confidence intervals between brackets

# Supplementary Table S3. Frequency of intermetatarsal bursitis, submetatarsal bursitis, Morton’s neuroma and submetatarsal diffuse alterations per location in patients with rheumatoid arthritis (RA), other arthritides and healthy controls.

|  | RA | Other arthritides | Healthy controls |
| --- | --- | --- | --- |
|  | **n (%)** | **n (%)** | **n (%)** |
| Intermetatarsal bursitis |  |  |  |
| - IT 1 | 59 (38) | 43 (18) | 15 (8) |
| - IT 2 | 70 (45) | 44 (16) | 9 (5) |
| - IT 3 | 90 (57) | 56 (20) | 21 (11) |
| - IT 4 | 32 (20) | 14 (5) | 3 (2) |
| Submetatarsal bursitis |  |  |  |
| - ST 1 | 20 (13) | 5 (2) | 2 (1) |
| - ST 2 | 11 (7) | 2 (1) | 1 (1) |
| - ST 3 | 7 (5) | 2 (1) | 0 (0) |
| - ST 4 | 5 (3) | 0 (0) | 0 (0) |
| - ST 5 | 18 (12) | 10 (4) | 4 (2) |
| Morton’s neuroma |  |  |  |
| - IT 1 | 8 (5) | 3 (1) | 1 (1) |
| - IT 2 | 15 (10) | 3 (1) | 1 (1) |
| - IT 3 | 31 (20) | 10 (4) | 4 (2) |
| - IT 4 | 1 (1) | 1 (0) | 0 (0) |
| Submetatarsal diffuse alterations |  |  |  |
| - ST 1 | 11 (7) | 19 (7) | 20 (10) |
| - ST 2 | 17 (11) | 23 (8) | 9 (5) |
| - ST 3 | 19 (12) | 24 (9) | 8 (4) |
| - ST 4 | 18 (12) | 13 (5) | 5 (3) |
| - ST 5 | 9 (6) | 16 (6) | 19 (10) |

RA: rheumatoid arthritis; IT: intermetatarsal space; ST: submetatarsal space.

# Supplementary Table S4. Test characteristics for rheumatoid arthritis (RA) determined by using cut-off points for transverse and dorsplantar diameters for intermetatarsal bursitis, submetatarsal bursitis and Morton’s neuroma.

|  | RA | RA vs. other arthritides | RA vs. healthy controls |
| --- | --- | --- | --- |
|  | Sensitivity, % | Specificity, % | Specificity, % |
| Intermetatarsal bursitis |  |  |  |
| - Transverse diameter ≥4mm | 13 (20/157) | 97 (9/284) | 98 (4/193) |
| - Dorsoplantar diameter ≥15mm | 16 (25/157) | 95 (14/284) | 100 (0/193) |
| Submetatarsal bursitis |  |  |  |
| - Transverse diameter^a^ | NA | NA | NA |
| - Dorsoplantar diameter ≥8mm | 13 (20/157) | 98 (5/284) | 100 (0/193) |
| Morton’s neuroma |  |  |  |
| - Transverse diameter ≥7mm | 12 (19/157) | 99 (3/284) | 100 (0/193) |
| - Transverse diameter ≥5mm | 16 (25/157) | 99 (4/284) | 97 (6/193) |
| - Dorsoplantar diameter ≥4mm | 13 (20/157) | 98 (6/284) | 100 (0/193) |

Cut-offs are based upon histograms: when a diameter was infrequent in the control groups it was used as a cut-off point.

^a^: No cut-off could be determined for the transverse diameter as there was overlap regarding all sizes, as is visualized in the histograms in Figure 4. NA: not applicable.

# REFERENCES SUPPLEMENTARY FILE

1. Ostergaard M, Peterfy C, Conaghan P, et al. OMERACT Rheumatoid Arthritis Magnetic

Resonance Imaging Studies. Core set of MRI acquisitions, joint pathology definitions, and the

OMERACT RA-MRI scoring system. *J Rheumatol*. 2003;30(6):1385-6.

2. Haavardsholm EA, Ostergaard M, Ejbjerg BJ, et al. Introduction of a novel magnetic

resonance imaging tenosynovitis score for rheumatoid arthritis: reliability in a multireader

longitudinal study. *Ann Rheum Dis*. 2007;66(9):1216-20.

3. Dakkak YJ, Matthijssen XME, van der Heijde DM, et al. Reliability of Magnetic Resonance

Imaging (MRI)-scoring of the Metatarsophalangeal-joints of the Foot According to the Rheumatoid

Arthritis-MRI Score (RAMRIS). *J Rheumatol*. 2019.

4. Mayerhoefer ME, Breitenseher MJ, Kramer J, Aigner N, Norden C, Hofmann S. Stir vs. T1-weighted fat-suppressed gadolinium-enhanced mri of bone marrow edema of the knee: Computer-assisted quantitative comparison and influence of injected contrast media volume and acquisition parameters. J Magn Reson Imaging 2005;22:788-93.

5. Schmid MR, Hodler J, Vienne P, Binkert CA, Zanetti M. Bone marrow abnormalities of foot and ankle: Stir versus t1-weighted contrast-enhanced fat-suppressed spin-echo mr imaging. Radiology 2002;224:463-9.

6. Stomp W, Krabben A, van der Heijde D, Huizinga TW, Bloem JL, van der Helm-van Mil AH, et al. Aiming for a shorter rheumatoid arthritis mri protocol: Can contrast-enhanced mri replace t2 for the detection of bone marrow oedema? Eur Radiol 2014;24:2614-22.

7. Sudol-Szopinska I, Jurik AG, Eshed I, Lennart J, Grainger A, Ostergaard M, et al. Recommendations of the essr arthritis subcommittee for the use of magnetic resonance imaging in musculoskeletal rheumatic diseases. Semin Musculoskelet Radiol 2015;19:396-411.

8. Dakkak YJ, Jansen FP, DeRuiter MC, et al. Rheumatoid Arthritis and Tenosynovitis at the

Metatarsophalangeal Joints: An Anatomic and MRI Study of the Forefoot Tendon Sheaths. *Radiology*.

2020:191725.

9. Zanetti M, Strehle JK, Zollinger H, et al. Morton neuroma and fluid in the intermetatarsal

bursae on MR images of 70 asymptomatic volunteers. *Radiology*. 1997;203(2):516-20.

10. Redd RA, Peters VJ, Emery SF, et al. Morton neuroma: sonographic evaluation. *Radiology*. 1989;171(2):415-7.
